# Prediction of peptide drift time in ion mobility mass spectrometry from sequence-based features

**DOI:** 10.1186/1471-2105-14-S8-S9

**Published:** 2013-05-09

**Authors:** Bing Wang, Jun Zhang, Peng Chen, Zhiwei Ji, Shuping Deng, Chi Li

**Affiliations:** 1 The Advanced Research Institute of Intelligent Sensing Network, Tongji University, shanghai, 201804, China; 2 The Key Laboratory of Embedded System and Service Computing, Ministry of Education, Tongji University, Shanghai 201804, China; 3 School of Electronics and Information Engineering, Tongji University, Shanghai 201804, China; 4 School of Electronic Engineering & Automation, Anhui University, Hefei, Anhui 230601, China; 5 Computer, Electrical and Mathematical Sciences and Engineering Division, King Abdullah University of Science and Technology, Thuwal, 23955-6900, Saudi Arabia; 6 Department of Medicine, University of Louisville, Louisville, KY 40202, USA

## Abstract

**Background:**

Ion mobility-mass spectrometry (IMMS), an analytical technique which combines the features of ion mobility spectrometry (IMS) and mass spectrometry (MS), can rapidly separates ions on a millisecond time-scale. IMMS becomes a powerful tool to analyzing complex mixtures, especially for the analysis of peptides in proteomics. The high-throughput nature of this technique provides a challenge for the identification of peptides in complex biological samples. As an important parameter, peptide drift time can be used for enhancing downstream data analysis in IMMS-based proteomics.

**Results:**

In this paper, a model is presented based on least square support vectors regression (LS-SVR) method to predict peptide ion drift time in IMMS from the sequence-based features of peptide. Four descriptors were extracted from peptide sequence to represent peptide ions by a 34-component vector. The parameters of LS-SVR were selected by a grid searching strategy, and a 10-fold cross-validation approach was employed for the model training and testing. Our proposed method was tested on three datasets with different charge states. The high prediction performance achieve demonstrate the effectiveness and efficiency of the prediction model.

**Conclusions:**

Our proposed LS-SVR model can predict peptide drift time from sequence information in relative high prediction accuracy by a test on a dataset of 595 peptides. This work can enhance the confidence of protein identification by combining with current protein searching techniques.

## Background

Ion mobility spectrometry (IMS) has gained significant attentions over the past few decades for rapid, high-resolution separations power, which can separate ions on a millisecond time-scale [1-3]. As a separation technique which based on differences in size and shape of analytes, IMS has proven powerful in the fields of metabolomics, glycomics and proteomics [1,2]. Ion mobililty-mass spectrometry (IMMS), an analytical technique by which IMS coupled with mass spectrometry (MS), have emerged as powerful tools for analyzing biological mixtures, especially for current proteomics studies [4-7]. By combination of the advantages of IMS and MS, IMMS opens up avenues for the detailed structural analysis of large and heterogeneous protein complexes, providing information on the stoichiometry, topology and cross section of their composition [8,9].

A typical proteomics experimental setup using IMMS consists of five components: sample introduction, compound ionization, ion mobility separation, mass separation as well as peptide and protein ion detection [10]. Although these five components all play essential roles in the process, ion mobility separation is crucial for its impact on the consequent mass analysis and peptide ion detection [11]. Ion mobility separation, by which the peptide ions with different cross-sections and molecular charges will be separated, adds a new dimension of separation and makes IMMS an attractive method for analyzing complex proteomics samples. Peptide ion separation can be enhanced by changing different gases, altering electric field strengths, and adopting non-linear electric field gradients, by which peptide identification can be facilitated to achieve high confidence [12]. Even though these efforts improve the separation capability of IMMS, they are still time-consuming, and it is difficult to reproduce under different experimental conditions.

Although IMMS separates peptide ions based on differing cross-sections and molecular charge, the experimental measurement behaves in the way that peptides spend different time through the drift tube. It has been reported that the measurement of peptide ion drift time using IMMS is very reproducible [13-18]. Any two measurements of mobilities (or cross sections) recorded on the same instrument usually agree to within 1% relative uncertainty. Measurements performed by different groups usually agree to within 2%. As a characteristic of different ions, peptide ion drift time can be used to enhance confidence in protein identifications.

There are several efforts which attempt to computationally determine the mobile behaviour of peptide ions in IMS. Valentine et al. predict peptide ion cross sections using intrinsic size parameters (ISPs) and tested it on 271 singly-charged peptides [19]. A quantitative structure-property relationship (QSPR) based approach was proposed for prediction of peptide drift time by Liu et al. and found the structure effect and the charge states of peptide ion contribute a lot to the drift time [20]. Shah et al. employed partial least squares (PLS) and support vector regression (SVR) based approaches to predict the drift time of massive peptide ions with different charge states and demonstrated both techniques significantly outperform the ISPs based calculation by a test on a high confidence database of 8,675 peptide sequences [21]. Zhang et al. presented a quantitative structure-spectrum relationship (QSSR) study to predict peptide drift time and found the sequence-based approach can get better fitting ability and predictive power but worse interpretability than the structure-based approach [22]. Our previous works also attempted to address the same problem by employing artificial neural networks and multiply linear regression models [23-25]. Although these studies contributed the drift time prediction of peptide ions a lot, ISP based calculations did not show the high performance in peptides with high charged states, and structure-based methods have to construct and optimize the geometrical structures of peptides which will bring inevitable errors into prediction models.

In this paper, a least square-support vectors regression (LS-SVR) model is presented to predict peptide ion drift time in IMMS just from the sequence-based features of peptide. The sequence pattern of each peptide was represented as a 36-component vector, which was consisted of for descriptors, i.e., molecular weight, sequence length, amino acid composition and pseudo amino acid composition. In construction of the LS-SVR regression, a 10-fold cross-validation strategy was employed to determine the optimized values of the regression parameters. Our proposed LS-SVR method was applied into three peptide ions datasets with different charge states, i.e., +1, +2, +3.

## Results and discussion

In this work, all the raw data generated from the IMMS were processed using MassLynx V4.1, an instrument control software, to obtain the drift time for each peptide ion peak. MassLynx is a powerful software for analyzing and processing the data acquired from mass spectrometers which are developed Waters Corporation. The peptides generated from tryptic digestion of 20 pure proteins were used for our model development and testing in this study. Peptide charge status was manually assigned based on the m/z spacing between isotopic peaks. As a result, the total of 595 peptides assigned ions which came from the 20 proteins became the dataset for this work. Within this dataset, 212 peptides were singly charged, 306 were doubly and 77 were triply charged. More details can be found in our previous work [12,26].

IMS separate ions based on the fact ions with different shapes and charge states travel though the drift tube at different velocities. In the drift tube, the ions were pulled by a weak electric field and opposed by the inset buffer gas. The charge state is a very important factor for the drift time. Therefore, we developed the SVR models for singly-, doubly- and triply-charged peptides, respectively. In this work we denotes dataset of singly-charged peptides as DataS, doubly-charged peptides as DataD, and triply-charged as DataT.

Table 1 shows the distributions of peptide molecular weight, sequence length and drift time in each of the three datasets. It can be seen that the smallest peptide just formed by 3 amino acids with singly-charge state, and the largest one have 34 amino acids from DataD and DataT, which indicate that peptides with large molecular weight and long amino acid sequences, tend to have high charge states. The peptide ion drift time is also significantly related to the overall ion charge state. The mean value of peptide drift time for the singly-charged peptides is 7.48 s while that of the doubly-charged and the triply-charged peptides are 3.07 s and 2.28 s, respectively. The peptides with high charge states drift through the cell in a relative high velocity. Another reason is the higher charge states the peptide is, the higher probability that they form a 3-dimensional spatial structure will be.

**Table 1:** Distribution of peptide molecular weight, sequence length and drift time in original datasets with different charge states

	Molecular weight (Da)		Sequence length		Drift time (s)	
	
	range	mean	range	mean	range	Mean
DataS	374.28-2088.9	900.14	3-19	7.9	2.17-24.5	7.48
DataD	605.35-3412.7	1470.39	5-34	13.2	1.08-9.39	3.07
DataT	981.56-3503.7	2046.30	8-34	18.3	1.08-3.97	2.28

## Prediction performance evaluation

In this study, we developed the LS-SVR models for predicting peptide drift time for the singly-, doubly-, and triply-charged peptides, respectively. A 10-fold cross-validation strategy was employed in the training and test process of the regression models, by which all observations in each datasets are used for both training and validation. This cross-validation can provide reliable learning of our model from the original data.

The purpose of this work is to predict ion drift time of peptides by elucidating the relationship between the dependent variable, i.e., peptide drift time, and the sequence-based peptide features we used, i.e., peptide molecular weight, sequence length, AAC and PseAAC. For regression analysis, there are many criteria by which they can be evaluated and compared. The root mean square error (*RMSE*) and coefficient of determination (*R*^2^) are selected in this work to evaluate the predictive performance of our LS-SVR models.

(1) $$RMSE=\sqrt{\frac{\overset{n}{\underset{i=1}{\sum }}{\left(dt_{i}-dt_{i}^{\prime }\right)}^{2}}{n}}$$

(2) $$R^{2}=1-\frac{\overset{n}{\underset{i=1}{\sum }}{\left(dt_{i}-dt_{i}^{\prime }\right)}^{2}}{\overset{n}{\underset{i=1}{\sum }}{\left(dt_{i}^{\prime }-{\bar{dt}}_{i}\right)}^{2}}$$

where *n *is the number of peptide in the dataset, *dt *is the experimentally observed peptide ion drift time, *dt *the predicted drift time by LS-SVR models, $\bar{dt}$ is the overall average value of peptide drift time. *R*^2 ^takes any value between 0 and 1, with a value closer to 1 indicating the regression model is of better performance.

Furthermore, in order to assess the prediction accuracy of LS-SVR models, a prediction variation threshold, *η_t_*, was defined by the relative variation of the predicted drift time from the experimentally observed values. If the relative variation between observed and predicted drift time is smaller than *η_t_*, the prediction will be seen as reliable, otherwise, unreliable.

(3) $$\eta =\frac{\left|dt-dt^{\prime }\right|}{dt}$$

Where *η *is the prediction variation, *dt*' is the predicted peptide ion drift time and *dt *is the experimentally observed peptide ion drift time.

## Parameters selection

As what state in Methods part, LS-SVR models with Gaussian kernel was adopted to predict peptides drift time. There are two important parameters for this kind of regression model, i.e., the width of Gaussian kernel parameter *σ*, and the regularization factor *γ*. The correct setting of these two parameters of the LS-SVR models is of critical importance in enabling us to achieve good regression performances. In this work, the grid-searching scheme is used to determine these two parameters based on cross validation strategy. Specifically, the *σ*^2 ^and *γ *were tuned simultaneously in a grid ranging from 2^-5^, 2^-4^, ..., 2^15 ^for *σ*^2 ^and from 2^-5^, 2^-4^, ..., 2^9 ^for *γ*. The prediction accuracy of LS-SVR models for each peptide dataset was seen as the objective function to determine the optimum combination of *σ*^2 ^and *γ*, where the value of *η_t _*was set as 0.15.

**Figure 1:**
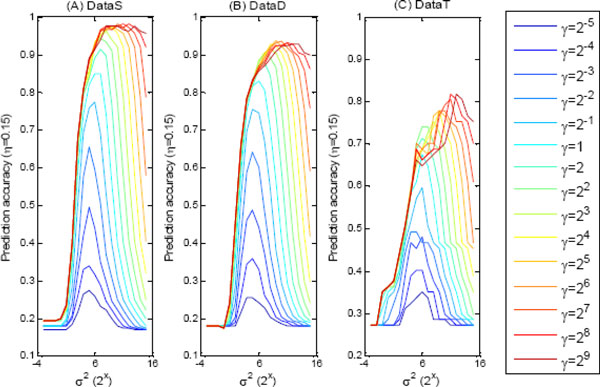
**Prediction accuracy curves of LS-SVR models in three peptide ion datasets when *η_t _*= 0.15, where *σ*^2 ^ranges from 2^-5 ^to 2^15 ^and *γ *ranges from 2^-5 ^to 2^9^**. (A) DataS, (B) DataD and (C) DataT.

The accuracy curves for different combination of the *σ*^2 ^and *γ *in the three peptide datasets were shown in the Figure 1. It can be seen that the regression performance of LS-SVR models are heavily depend on the selection of the parameters *σ*^2 ^and *γ*. When *γ *is fixed, the prediction accuracy goes up with the increase of *σ*^2 ^to an apex and then goes down. For DataS, the top 5 prediction accuracy values correspond to the combinations [*σ*^2^, *γ*] of [2^10^, 2^6^], [ 2^11^, 2^7^], [ 2^12^, 2^8^], [ 2^13^, 2^9^], and [2^9^, 2^5^]. The top 5 LS-SVR models for DataD have the combination parameters of [2^9^, 2^5^], [2^10^, 2^6^], [ 2^11^, 2^7^], [ 2^11^, 2^8^], and [2^9^, 2^6^]. For the peptide dataset with triply-charge, DataT, the top 5 combinations are [2^11^, 2^8^], [2^12^, 2^9^], [2^10^, 2^7^], [2^11^, 2^8^], and [2^12^, 2^9^]. Overall the three datasets, the value [2^11^, 2^8^] can achieve the best prediction accuracy for the LS-SVR models when *η_t _*= 0.15. Therefore, the *σ*^2 ^of 2^11 ^and *γ *of 2^8 ^were selected for the subsequent analysis in this work.

## Prediction performance

A 10-fold cross validation was implemented in the construction of LS-SVR models, by which the different separation of the original dataset will bring the changes of predicted drift time for each peptide. For evaluating the uncertainty in the regression performance of our model which come from the randomness of the dataset separations, the regression procedure was repeated for ten times. The mean of the prediction drift times for each peptide from these ten times experiments were taken as the finally predicted value. Also the variation of the ten times was studied to exam the stability of our proposed LS-SVR models.

The prediction performance was shown in Table 2. It can be seen that our models ca achieved very good prediction ability for different peptide dataset, i.e., 0.9811 for DataS, 0.9379 for DataD, and 0.8312 for DataT. Comparing to DataS and DataD, the prediction accuracy of the triply-charge peptide ions in DataT is a little bit poor. One reason for this situation is that the dataset's size is small, i.e., 77 peptide in DataT, which can not provide sufficient information in the model training. Another reason, we believe, is that the charge state of DataT is higher than that of DataS and DataD, which usually cause the peptide longer. The mean length of peptides in DataT is 18.3, which is 1.4 times of that in DataD, and 2.3 times in DataS. The longer of the peptide length is, the more chance the peptide form the secondary structure will be. Obviously, the changes in space conformation will contribute the peptide's velocity in drift cell and therefore, affect the peptide ion's drift time.

**Table 2:** Prediction performance of LS-SVR models under a variation threshold of 15% in three peptide ion's datasets

	Prediction accuracy^a^		RMSE	R^2^
DataS	0.9811	(0.9736±0.081)	0.5202	0.9718
DataD	0.9379	(0.9340±0.061)	0.2602	0.9721
DataT	0.8312	(0.7883±0.025)	0.2637	0.8727

a. The prediction accuracy for each dataset was shown as the format of A(B±C), where A denotes the prediction accuracy from the mean of predicted drift times, B the mean prediction accuracy of the ten repeat times, and C the standard deviation of the accuracy of the ten repeat times.

It can be found from Table 2 that the prediction accuracy from the mean of the predicted drift times is better than the mean accuracy of the ten repeat experiments. It can get 0.0075, 0.0039 and 0.0479 for DataS, DataD, and DataT, respectively, which indicated that the combination regression model will improve the predictive power of predictors. From Table 2, it can also be seen that the standard deviation of the prediction accuracy of the ten repeat experiments is very small, i.e., 0.081, 0.061 and 0.025 for the three datasets. It demonstrate our LS-SVR models are stable and statistically valid because a small change in the data, such as the different split of the training and test dataset, may lead to large changes of the prediction performance.

**Figure 2:**
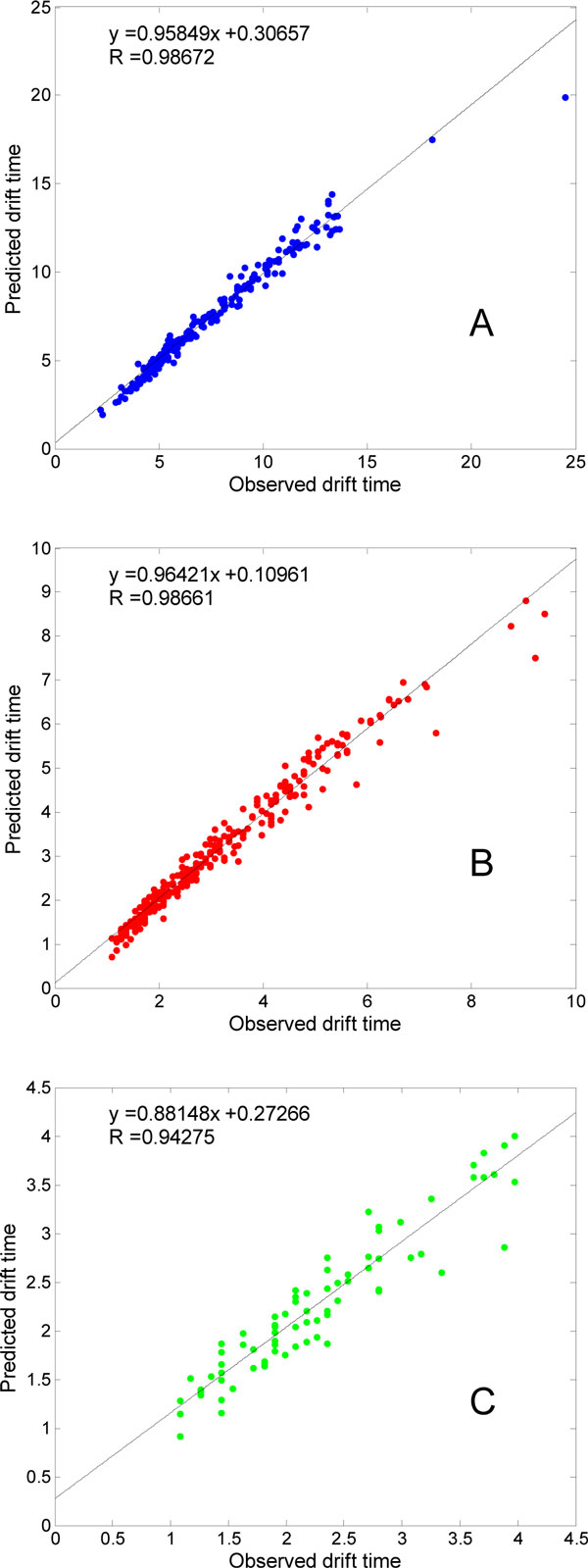
**Regression performance between the observed and predicted drift times for the peptide ions with different charge states**. **(A) DataS, (B) DataD, and (C) DataT**. The linear function in each subfigure is the linear fitted function between the observed and predicted drift time for every datapoint in each dataset, and the line is the corresponding fitted curve. *R *denotes the correlation coefficient of observed vs. predicted drift time.

The relative small *RMSE *and *R*^2 ^shown in Table 2 also indicted the powerful regression performance of LS-SVR models in prediction of peptide ion's drift times in IMMS. We got very small *RMSE *values for DataD and DataT, and a little higher value, 0.52, for DataS, which is reasonable for the big range of the original drift time, from 2.17 s to 24.5 s. The *R*^2 ^values of around 0.97 for DataS and DataD, 0.87 for DataT are shown high correlation between the predicted and experimental observed peptide drift times. More details about the regression results can be found in Figure 2, where the line showed the linear fitting between the predicted and observed drift time in a least-squares sense. The high correlation coefficients, i.e., 0.987 for both DataS and DataD, and 0.943 for DataT, signifies the LS-SVR model we proposed here can capture the general properties by which different peptides fly through drift cell in different velocities.

**Figure 3:**
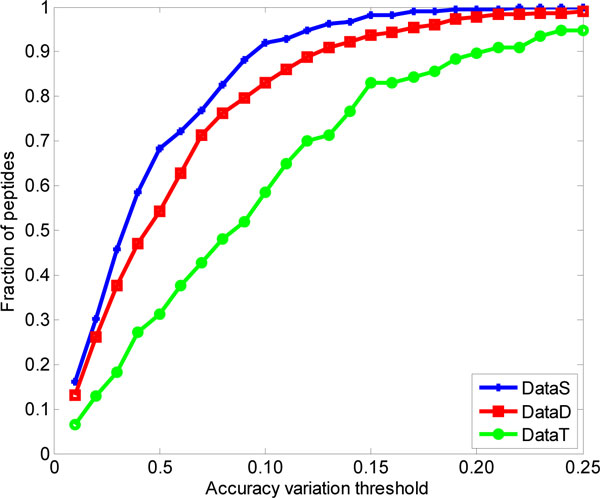
**Fraction of peptide ions correctly predicted at different accuracy variation levels**. A higher curve indicates a larger number of peptides for a given threshold value.

After the LS-SVR models had finished the regression analysis for the three datasets with different charge states ions, the variation threshold *η_t _*will decide which peptide can be predicted correctly. Figure 3 displays the relation between the fraction of peptide ions whose drift time are predicted correctly and the accuracy threshold *η_t_*. It can be seen that our proposed method can get best prediction performance in the DataS. The reason we believe is the peptides in DataS are small and have higher probability they adopt elongated conformations in order to minimize coulomb repulsion, while the peptides in DataT usually are large and have higher probability to form secondary structure when they go through the drift cell in IMMS instrument. It can be found even the variation threshold is set as 0.10, there are more than 90% peptides can be predicted correctly, by which the prediction performance of our LS-SVR model can be demonstrated. If the conformation information can be added into the regression model, the predictive power for doubly- and triply-charge peptides will be increased undoubtedly.

## Conclusions

To enhance the confidence of peptide identification, a LS-SVR model was developed in this study to predict peptide ion drift time for IMMS measurements. In LS-SVR, there are two parameters, i.e., the width of Gaussian kernel parameter *σ*, and the regularization factor *γ*, have to be selected for their influence on the regression accuracy. A grid searching strategy was employed to optimize the selection of these two parameters. Based on the peptide sequence, a 34-component vector was extracted as representation to construct our LS-SVR models on three peptide ion datasets with different charge states. With the prediction accuracy threshold *η *was set to 0.15, we achieved very high performance, i.e., 0.9811 and 0.9379, for the peptide ions with singly- and doubly-charge, which indicated the prediction capability of the LS-SVR models. It is reasonable that there is a relative lower prediction accuracy of 0.8312 for DataT, for the peptides with higher charge states have a higher probability that they can form a secondary structure. This kind of situation will be improved if the structure information can be added into our proposed LS-SVR models; even more computational cost will be requested.

## Methods

### Peptide dataset

The total of 595 peptides of 20 pure proteins used in this work was reported in our previous work [12]. The proteins were purchased from Sigma Aldrich and used without further purification. The peptide fragments were produced from the pure proteins according to the details of the sample preparation section in the report, and then were analyzed by direct electrospray into the Synapt HDMS instrument (Waters). Peptide ion assignments were obtained from a peptide mass fingerprint for each tryptic digest. As a result, in the dataset with 595 peptide ions, there are 212 peptides were singly charged, 306 were doubly charged and 77 were triply charged. More details about the experimental processing of samples can be obtained from the work [12,26].

### Support vector regression

Support vector machines, a specific class of machine learning algorithms which was firstly proposed by Vapnik and his co-workers in 1995 [12], have proven very effective for solving pattern classification problems, even for the dataset in small size. For a binary classification problem, the main idea of SVM is to select a hyper-plane that separates the positive from negative samples while maximizing the minimum margin. Currently, SVM has been became one of the most popular machine learning methods, which has been applied to various domains of interest, such as bioinformatics, cheminformatics, image processing, data mining, knowledge discovery, and etc. In many applications, SVM can achieve excellent performance for the character that the capacity of the SVM system is controlled by parameters that do not depend on the dimensionality of feature space [27-32].

In the same way as with classification task, SVM can also be applied to the case of regression which is called support vector regression (SVR). In statistics, regression analysis is a statistical technique for estimating the relationships among variables. All the regression tasks can be formulated as to seek an estimation function which can approximate the observations within an acceptable error range. In this study, least square support vector regression (LS-SVR), a version of SVR which can reduce the complexity of optimization processes, was adopted for the drift time prediction[33].

Given a training dataset *D *= {*x_i_*, *y_i_*}(*i *= 1, 2, ..., *n*), *x ***R **∈ ^n ^, *y *∈ **R**, where *x_i _*is the input vector, *y_i _*is its corresponding target vector and *n *is the size of the dataset, SVR can construct regression model by using nonlinear mapping function *ϕ*(·) as follows:

(4) $$y\left(x\right)=w^{T}\phi \left(x\right)+b,w\in x,b\in R$$

where *w *is the vector of coefficients and *b *a constant. Usually, *w *and *b *are obtained by minimizing the upper bound of generalization error. Accordingly, the regression problem in LS-SVR can be transformed into the following optimization problem[34]:

(5) $$\begin{array}{c} \text{min}\;1/2w^{T}w+1/2\gamma \overset{l}{\underset{i=1}{\sum }}\overset{2}{\underset{i}{e}}\\ s.t.\;\;y_{i}=w^{T}\phi \left(x_{i}\right)+b+e_{i}\left(i=1,2,\ldots ,l\right) \end{array}$$

where *γ *is the regularization parameter, is applied to control the minimization of estimation error and the function smoothness, and *e_i _*is the error between actual output and predictive output of the *i *-th input data. The high value of *γ *denotes the good fitting of the training data points is stressed, and in the case of noisy data a smaller *γ *value should be taken to avoid overfitting. In order to solve the optimization problem, the Lagrangian function is formulated as following:

(6) $$L\left(w,b,e,\alpha \right)=1/2w^{T}w+1/2\gamma \overset{n}{\underset{i=1}{\sum }}e_{i}^{2}-\overset{n}{\underset{i=1}{\sum }}\alpha_{i}\left[w^{T}\phi \left(x_{i}\right)+b+e_{i}-y_{i}\right]$$

where *α *= (*α*_1_, *α*_2_, ..., *α_l_*) is the Lagrange multiplier. The KKT conditions are used for optimality by differentiating *L *with the variable *w*,*b*,*e*,*α*, which is shown as follows.

(7) $$\left\{\begin{array}{l} \frac{\partial L}{\partial w}=0\to w=\overset{n}{\underset{i=1}{\sum }}\alpha_{i}\phi \left(x_{i}\right)\\ \frac{\partial L}{\partial b}=0\to \overset{n}{\underset{i=1}{\sum }}\alpha_{i}=0\\ \frac{\partial L}{\partial e_{i}}=0\to \alpha_{i}=\gamma e_{i},\;i=1,\ldots ,n\\ \frac{\partial L}{\partial \alpha_{i}}=0\to w^{T}\phi \left(x_{i}\right)+b+e_{i}-y_{i}=0,\;i=1,\ldots ,n \end{array}\right)$$

By solving the upper linear system, the final solution of the primal problem can be represented in the following form.

(8) $$f\left(x\right)=\overset{n}{\underset{i=1}{\sum }}w_{i}K\left(x,x_{i}\right)+b$$

where *K*(•) is kernel function which can satisfy Mercer's condition corresponds to a dot product ion some feature spaces [34]. The most used kernel functions include the Gaussian RBF *K*(*x*, *x_i _*) = exp(||*x − x_i_|| */ 2*σ*^2^) with a width of *σ*, sigmoid and the polynomial kernel *K*(*x*, *x_i_*) = (*a*_1_*xx_i_+a*_2_)*^d ^*with an order of and constants *a*_1 _and *a*_2_. Gaussian RBF kernel is employed in this study, and the kernel parameter *σ*2 and *γ*, therefore, should be determined firstly. Currently, many approaches have been applied in parameter optimization of SVR, such as experience [27], grid searching [35], particle swarm optimization(PSO) [36], genetic algorithm(GA) [37], simulated annealing algorithm [38]. Considering computing complexity, cross-validation grid searching, the most used method, is selected to determine the parameters *σ*2 and *γ *in LSSVR model.

### Peptide representation

To implement LS-SVR model to predict peptide drift time in IMMS, each peptide have be represented as a vector with specific peptide features. Because each peptide is not consistent in the length, and the shape is affected by the charge state of the peptides, only features were extracted from the peptide sequence, therefore, are used to represent the peptide in this work.

### Peptide molecular weight

In IMMS, the ions are pulled by a uniform electric field through the buffer gas in the drift cell. Therefore, the molecular weight of peptide is one of the most important parameters which can affect ion mobility. Karasek et al. found there is a linear relationship between the reduced mobility of alkylamines and molecular weight under a specific experimental setting [39]. Also, other researches reported that the reduced mobility is inversely proportional to ion mass [40]. For a peptide *P *whose sequence is consisted of *N *amino acid residues as follows:

(9) $$P=R_{1}R_{2}\cdots R_{i}\cdots R_{N}$$

Where *R_i _*denote the *i *-th amino acid in the peptide. The molecular weight of *P *can be calculated as:

(10) $$MW_{P}=\overset{N}{\underset{i=1}{\sum }}mw_{i}+\left(N-1\right)\times 18$$

where *mw_i _*is the molecular weight of *i *-th amino acid in the peptide sequence.

### Sequence length

The sequence length (*SL*) of peptide, *N*, plays an important role in the formation of peptide's structure. The longer of the peptide sequence is, the more chance the peptide folds into a secondary or tertiary structure. Except charge states, IMS distinguishes ions based on the ion shapes which is affected by the sequence length. The previous work indicated that peptides only with primary structure will have smaller ion mobility than that with secondary structure, and smaller more than that with tertiary structure.

### Amino acid composition

All the peptide information is contained in its complete amino acid sequence. Therefore, it is the best choice for representing each peptide by its complete sequence. Amino acid composition (AAC) is one of the popular approaches to address protein or peptide representation problem because it is simple, yet powerful feature in prediction of protein structure, interaction, and functional sites. Generally, there are only twenty standard amino acid residues are considered in AAC. Therefore, AAC is a 20-components vector, where each component shows the occurrence number of an amino acid type in the peptide sequence (in many works, ACC is expressed by the occurrence frequencies, not numbers). For peptide *P*, ACC can be expressed by

(11) $$ACC_{P}={\left(a_{1}\;a_{2}\;...\;a_{20}\right)}^{T}$$

Where *a_i _*denotes the normalized frequency of *i *-th type of amino acid in peptide *P*.

### Pseudo-amino acid composition

Though AAC can represent peptides in a very simply way, it ignores all the information of amino acid sequence-order effects, which decide the local environment of each amino acid in the peptide. Therefore, Pseudo amino acid composition (PseAAC) was originally introduced by Kuo-Chen for representing proteins and had demonstrated its effectiveness in improving protein subcellular localization prediction, membrane protein type prediction and other works [41]. For peptide *P*, PseAAC could be formulated as

(12) $$PseAAC_{P}={\left(p_{1},\;\;p_{2},\;\;\cdots \;,\;\;p_{20},\;\;p_{20+1},\;\;\cdots \;,\;\;p_{20+\lambda }\right)}^{T},\left(\lambda <N\right)$$

Where *p*_1_, *p*_2_, ..., *p*_20_, are associated with the conventional amino acid composition of *P*, which already represented by sequence length and ACC in above, and $p_{20+1},p_{20+2},\cdots \;,p_{20+\lambda }$ are the *λ *correlation factors that reflect the 1st tier, 2nd tier, ..., and the *λ*-th tier sequence order correlation patterns. Therefore, only $p_{20+1},p_{20+2},\cdots \;,p_{20+\lambda }$ in *PseAAC_P _*have been adopted for representing peptides. In this work, six characters of 20 amino acid, i.e., hydrophobicity, hydophilicity, mass, pK1(alpha-COOH), pK2(NH3) and pI(at 25 °), have been used for calculated *PseAAC_P _*, and *λ *is set up to 2.

### Feature normalization

From the above section, it can be found that four types of sequence-based features were applied to represent peptides. However, these four features are of different physical dimension of quantity and different value ranges. The imbalanced expression level of different features will result in a variation in contribution of each of them to the drift time predictor. To remove the bias of expression level, all of the feature values have to be normalized to equally reflect (as much as possible) the influence of each feature. In this work, all values of each feature always fall within a fixed interval [-1, 1] by

(13) $$f_{normalized}=2\times \left(f-f_{\text{min}}\right)/\left(f_{\text{max}}-f_{\text{mim}}\right)-1$$

where *f *is the raw value of feature, *f_normalized _*denotes the normalized value of this feature, *f*_min _and *f*_max _are the minimum and maximum values of the corresponding feature category.

### Regression model construction

In our experiment, regression predictor is designed using LS-SVR model to solve drift time prediction from peptide sequence-based features. Based on the description of peptide representation, the LS-SVR model for predicting peptide drift time are constructed on a vector consisted of four sequence-based features, of which *MW *is of with 1 dimension, *SL *with 1 dimension and *AAC *with 20 dimensions. For PseAAC, the dimension is 12 for we employed 2-tier sequence correlation pattern with 6 amino acid characters. As a result, each peptide is represented in the predictor by a 34-component vector. For the peptide datasets, i.e., DataS, DataD and DataT, we construct three LS-SVR model for each dataset because the determinative effect of charge state to ion mobility.

### Cross-validation

To evaluate the prediction performance of each regression model, a 10-fold cross-validation strategy was adopted for regression analysis. Specifically for singly-charged peptides, DataS is randomly partitioned into 10 sub-datasets, of which a single sub-dataset is retained as the validation data for testing the model, and the remaining 9 sub-datasets are used as training data. After training processes were finished, the LS-SVR model can be applied to the prediction task. This process is then repeated 9 times with each of the ten sub-datasets used exactly once as the testing data. The 10 results from the folds are combined to evaluate the prediction performance.

## Competing interests

The authors declare that they have no competing interests.

## Authors' contributions

BW and JZ conceived of the study; ZJ, SD and CL participated in the experiment design; BW, JZ and PC carried it out and drafted the manuscript. All authors revised the manuscript critically.

## Declarations

This article has been published as part of *BMC Bioinformatics *Volume 14 Supplement 8, 2013: Proceedings of the 2012 International Conference on Intelligent Computing (ICIC 2012). The full contents of the supplement are available online at http://www.biomedcentral.com/bmcbioinformatics/supplements/14/S8.
